# Pigment epithelium-derived factor alleviates endothelial injury by inhibiting Wnt/β-catenin pathway

**DOI:** 10.1186/s12944-017-0407-8

**Published:** 2017-02-07

**Authors:** Shouyuan Ma, Shutong Yao, Hua Tian, Peng Jiao, Nana Yang, Ping Zhu, Shucun Qin

**Affiliations:** 1 Department of Geriatric Cardiology, Chinese PLA General Hospital, Beijing, 100853 China; 2 Key Laboratory of Atherosclerosis in Universities of Shandong and Institute of Atherosclerosis, Taishan Medical University, Taian, Shandong 271000 China; 3 College of Basic Medical Sciences, Taishan Medical University, Taian, Shandong 271000 China

**Keywords:** Pigment epithelium-derived factor, Endothelial injury, Wnt/β-catenin pathway, Atherosclerosis, Oxidative stress, Oxidized low-density lipoprotein

## Abstract

**Background:**

Oxidized low-density lipoprotein (ox-LDL) can induce endothelial injury and plays a vital role in the procession and development of atherosclerosis. Little is known regarding whether Wnt/β-catenin pathway is involved in ox-LDL-induced endothelial injury or whether it further promotes atherosclerosis via increased oxidative stress. This study aimed to investigate the role of Wnt/β-catenin pathway in ox-LDL-induced vascular endothelial injury and determine whether pigment epithelium-derived factor (PEDF) could alleviate ox-LDL-induced endothelial injury by inhibiting Wnt/β-catenin pathway.

**Methods:**

Injury of human umbilical vein endothelial cells (HUVECs) was evaluated with an MTT assay, by monitoring lactate dehydrogenase (LDH) release and determining the apoptotic ratio. The expression of β-catenin (non-phosphorylated-β-catenin), disheveled-1 (Dvl-1) and Cyclin D1 was analyzed with western blotting and quantitative real-time PCR. Oxidative stress status was assessed by measuring the levels of reactive oxygen species (ROS), malondialdehyde (MDA), superoxide dismutase (SOD) and nitric oxide (NO).

**Results:**

Exposure of HUVECs to ox-LDL led to a decrease in cell viability and an increase in LDH release and apoptosis with concomitant enhancement of oxidative stress, as assessed by increased ROS and MDA generation, as well as decreased SOD activity and NO levels. Similar to lithium chloride (LiCl, a Wnt/β-catenin pathway activator), ox-LDL up-regulated the expression of β-catenin, Dvl-1 and Cyclin D1, markers of Wnt/β-catenin pathway activation. However, ox-LDL-induced activation of Wnt/β-catenin pathway, as well as ox-LDL-induced cell injury and oxidative stress, were synergistically promoted by LiCl and mitigated by Dickkopf 1 (DKK-1), an inhibitor of Wnt/β-catenin pathway. Additionally, ox-LDL-induced HUVEC injury and apoptosis, oxidative stress and activation of Wnt/β-catenin pathway were suppressed by PEDF, while they were further strengthened by a small interfering RNA of PEDF.

**Conclusion:**

Wnt/β-catenin pathway may mediate ox-LDL-induced endothelial injury via oxidative stress, and PEDF ameliorates endothelial injury by suppressing Wnt/β-catenin pathway and subsequently reducing oxidative stress.

**Electronic supplementary material:**

The online version of this article (doi:10.1186/s12944-017-0407-8) contains supplementary material, which is available to authorized users.

## Background

Cardiovascular disease (CVD) is ranked first in morbidity and mortality, accounting for nearly a third of deaths worldwide [1]. Atherosclerosis, characterized by chronic inflammation and lipid accumulation in the vascular wall, is believed to be the main cause of CVD [2–4]. Vascular endothelial injury and dysfunction is the first step in the initiation of atherosclerosis [5]. As endothelial cells (ECs) are injured, vascular homeostasis is impaired, leading to an increase in vascular permeability and lipid accumulation, up-regulation of adhesion molecules and inflammatory factors, and macrophage-derived and vascular smooth muscle cell (VSMC)-derived foam cells formation, which all contribute to the development of atherosclerosis [6]. Moreover, previous studies have proposed that oxidized low-density lipoprotein (ox-LDL) plays a critical role in the procession and development of atherosclerosis and ox-LDL may induce endothelial injury by enhancing endothelial activation, impairing endothelium-dependent vasorelaxation, increasing oxidative stress and causing EC apoptosis [7, 8]. However, the precise mechanism underlying ox-LDL-induced endothelial injury is still not fully clarified. Therefore, further elucidation of the mechanisms responsible for ox-LDL-induced endothelial injury will be crucial for the formation of novel therapeutic approaches to combat atherosclerotic lesion progression.

Wnt/β-catenin signaling pathway, a canonical Wnt pathway, is thought to be essential in a number of developmental and physiological processes [9]. Many lines of evidence have identified the vital role of Wnt/β-catenin pathway in CVD as well as in the progression of atherosclerosis [10, 11]. Bradley D has found that Wnt/β-catenin signaling preferentially occurs in regions predisposed to atherosclerosis and atheroprone hemodynamics promotes this pathway activation in mice [12]. Significant expression of active β-catenin was also demonstrated in human disrupted plaques [13]. Dickkopf 1 protein (DKK-1), a downstream target of Wnt/β-catenin signaling, is thought to be a novel biomarker of coronary atherosclerosis [14]. In addition, activation of Wnt/β-catenin signaling can enhance monocyte adhesion to ECs [15], cause endothelial dysfunction [16] and up-regulate inflammatory cytokines [17, 18]. Interestingly, oxidative stress can activate Wnt/β-catenin signaling [19, 20]; in turn, suppressing this signaling can block the production of reactive oxygen species (ROS) [21]. Furthermore, ox-LDL can promote Wnt/β-catenin signaling activation in human retinal pigment epithelium (RPE) cells and elevate the levels of ROS, tumor necrosis factor (TNF-α) and vascular endothelial growth factor (VEGF) and promote vascular calcification and the formation of foam cells [22–24]. It is reported that β-catenin-elicited oxidative stress mediates the apoptosis of VSMCs, which contributes to atherosclerotic plaque rupture [25]. Nevertheless, whether Wnt/β-catenin pathway is involved in ox-LDL-induced endothelial injury and further promotes atherosclerosis via increased oxidative stress still needs to be clarified.

Pigment epithelium-derived factor (PEDF) belongs to the serine protease inhibitor supergene family. As previously confirmed, PEDF is a type of endogenously produced multifunctional protein with anti-oxidative, anti-inflammatory, anti-angiogenic, anti-thrombotic, neuroprotective and neurotrophic properties [26, 27]. In addition, much work in the past has been done to determine the role of PEDF in CVD, especially in atherosclerosis [26–28]. Serum level of PEDF is believed to be a marker of atherosclerosis in humans [29, 30], and PEDF plays a protective role in atherosclerosis by inhibiting the inflammatory response and angiogenesis [27, 31, 32]. Thus, PEDF has attracted considerable interest as an anti-atherosclerotic and a therapeutic target for prevention and treatment of CVD. Interestingly, high levels of PEDF inhibited the activation of Wnt/β-catenin pathway in endothelial progenitor cells in diabetes [33]. Additionally, as an endogenous antagonist of low-density lipoprotein-related protein (LRP) 6, PEDF has been shown to suppress Wnt/β-catenin signaling in retinal cells [34]. Furthermore, we previously reported that PEDF was significantly lower in acute coronary syndrome (ACS) patients than in controls, and lower PEDF levels were further associated with adverse cardiac outcomes after ACS [35]. We also determined that D-4F, an apolipoprotein A-I mimetic peptide, effectively protected ECs against ox-LDL-induced injury by up-regulating PEDF [36], suggesting that PEDF may ameliorate ox-LDL-induced endothelial injury. However, it is still not clear whether PEDF could protect ECs against ox-LDL-elicited damage by blocking Wnt/β-catenin signaling. Therefore, in the present study, we aimed to investigate the role of Wnt/β-catenin pathway in ox-LDL-induced endothelial injury and assess whether PEDF could alleviate ox-LDL-induced injury by inhibiting Wnt/β-catenin pathway.

## Methods

### Reagents

Dulbecco’s modified Eagle medium (DMEM) and fetal bovine serum (FBS) were purchased from Gibco (BRL, Gaithersburg, MD, USA). Ox-LDL was purchased from Union-Biology (Beijing, China). DKK-1 and PEDF were obtained from PeproTech (Rocky Hill, NJ, USA). Lithium chloride (LiCl), 3-(4,5-dimethyl-2-thiazolyl)-2,5- diphenyl-2-H-tetrazolium bromide (MTT) dye and 2′,7′-dichlorofluorescin diacetate (DCFH-DA) were purchased from Sigma (St Louis, MO, USA). Annexin V-FITC/propidium iodide (PI) apoptosis detection kits were obtained from KeyGEN Biotech (Nanjing, China). SYTO-13/PI dye was purchased from Life Technologies (Carlsbad, CA, USA). Anti-β-catenin (non-phosphorylated β-catenin) polyclonal antibody, anti-disheveled-1 (anti-Dvl-1) polyclonal antibody and anti-PEDF polyclonal antibody were obtained from Santa Cruz Biotechnology (Santa Cruz, CA, USA). TRIZOL reagent, OPTI-MEM I reduced-serum medium and Lipofectamine 2000 were purchased from Invitrogen (Carlsbad, CA, USA). QuantScript RT kits and RealMaster-Mix (SYBR Green) kits were obtained from Tiangen Biotech (Beijing, China). Lactate dehydrogenase (LDH), malondialdehyde (MDA), superoxide dismutase (SOD) and NO assay kits were purchased from Nanjing Jiancheng Bioengineering Institute (Nanjing, China).

### Cell culture and treatment

A human umbilical vein endothelial cell (HUVEC) line was obtained from the Type Culture Collection of the Chinese Academy of Sciences (Shanghai, China). Cells were cultured at 37 °C in a humidified 5% CO_2_ atmosphere in DMEM supplemented with 5 mM glucose, 10% FBS and 1% penicillin-streptomycin until subconfluent, and then, the medium was replaced with DMEM containing 1% FBS.

HUVECs were treated with ox-LDL at different concentrations (12.5, 25, 50, 100 and 150 mg/L) for 24 h. In addition, cells were pretreated with 20 nM DKK-1 and 20 mM LiCl for 24 h, or with different concentrations (50, 100, 200 and 400 ng/mL) of PEDF for 24 h, followed by exposure to 100 mg/L ox-LDL for 24 h.

### Cell viability and LDH assay

Cell viability was determined using an MTT assay. HUVECs were seeded into 96-well plates at a density of 1 × 10^4^ cells/well. Following treatment, 20 μL of MTT dye was added to each well at a final concentration of 0.5 mg/mL and cells were incubated for 4 h at 37 °C. After removing the supernatant, 150 μL of dimethylsulfoxide (DMSO) was added and the optical density was measured at 490 nm using a microplate reader (Tecan Infinite F200, Switzerland). Cell viability is displayed as the percentage of the optical density of treated cells relative to that of the untreated control cells (100%).

To further determine the degree of cell injury, LDH activity in the medium was assessed using an assay kit according to the manufacturer’s instructions. LDH activity was measured at 450 nm and expressed as units per liter (U/L).

### Evaluation of cell apoptosis

The quantification of apoptosis was performed with an Annexin V-FITC/PI double-staining assay. Treated cells were centrifuged, harvested and washed with phosphate-buffered saline (PBS). After resuspension in 500 μL of binding buffer, cells were double-stained with 5 μL of Annexin V–FITC and 5 μL of PI at room temperature in the dark for 15 min. Then, the samples were analyzed using Cell-Quest software on a Becton Dickinson FAC Scan flow cytometer (Becton Dickinson, San Jose, CA, USA). The total apoptotic cells (early and late-stage apoptosis) were represented by Annexin V-FITC staining alone or together with PI.

Morphological changes of cell apoptosis were observed using SYTO-13/PI dye staining. Cells cultured on glass coverslips were fixed in 4% paraformaldehyde for 30 min and were washed 3 times. Subsequently, coverslips were stained with 0.99 μM SYTO-13 dye and 6.56 μg/mL PI dye diluted with PBS at room temperature in the dark for 15 min. Cells were visualized using an Olympus BX51 microscope (Olympus, Tokyo, Japan) and were analyzed with Image-Pro Plus software (version 6.0; Media Cybernetics, Bethesda, MD, USA).

### Western blotting

Treated cells were lysed with Radio Immunoprecipitation Assay (RIPA) lysis buffer containing 1% protease inhibitors to obtain the total protein. Equal amounts of protein were separated by 8 to 12% SDS-PAGE gels and were transferred onto polyvinylidene fluoride (PVDF) membranes by electroblotting. After being blocked with 10% fat-free dried milk in Tris-buffered saline (TBS) containing 0.1% Tween-20 for 4 h at room temperature, the membranes were incubated with primary antibody overnight at 4 °C. Antibody-treated membranes were washed with TBS containing 0.1% Tween-20 and then were incubated with horseradish peroxidase-conjugated secondary antibodies for 2 h at room temperature. Specific bands were visualized with an electrochemiluminescence (ECL) reagent. The integrated optical density of protein bands was assessed with Image-Pro Plus software and normalized to β-actin.

### Quantitative real-time PCR

Total RNA was isolated using TRIZOL reagent. Complementary DNA (cDNA) was synthesized from 2 μg of total RNA using a QuantScript RT kit according to the manufacturer’s protocol. Subsequently, a quantitative real-time PCR reaction was performed with a RealMaster-Mix (SYBR Green) kit, and products were amplified using a Rotor-Gene quantitative real-time PCR machine (QIAGEN, Germany). Primers used in this study were synthesized by Sangon Biotech (Shanghai, China). For human β-catenin, the primers were 5′-GTGTGGCGACATATGCAGCT-3′ (forward) and 5′-CAAGATCAGCAGTCTCATTC-3′ (reverse). For human Cyclin D1, the primers were 5′-GTGCATC TACACC GACAACTCCA-3′ (forward) and 5′-TGAGCTTGTTCACCAGGAGCA-3′ (reverse). For human GAPDH, the primers were 5′-CCTCCCGCTTC GCTCTCT-3′ (forward) and 5′-GCTGGCGACGCAAAAGA-3′ (reverse). For human PEDF, the primers were 5′-ATCGTCTTTGA GAAG-3′ (forward) and 5′-CAAACTTTGTTACCCACTGC-3′ (reverse). The data were analyzed using Rotor-Gene Q software (version 1.7; Qiagen). Relative mRNA levels were calculated using the 2^–△△Ct^ method and normalized against GAPDH.

### Detection of intracellular ROS generation

Intracellular ROS generation was measured with the peroxide-sensitive fluorescent probe 2′,7′-dichlorofluorescein diacetate (DCFH-DA) using flow cytometry. HUVECs cultivated in 6-well plates were incubated with 10 mM DCFH-DA in FBS-free DMEM at 37 °C for 30 min. Then, the cells were collected and analyzed on a FACScan flow cytometer using CellQuest software.

### Measurement of intracellular SOD activity, MDA content and NO level in medium

Intracellular SOD activity, MDA content and NO levels in the medium were detected using assay kits according to the manufacturer’s instructions and expressed as units per milligram of protein (U/mgprot), nanomoles per milligram of protein (nmol/mgprot) and micromoles/liter (μM).

### PEDF-siRNA transfection

HUVECs were cultured in antibiotic-free growth medium to reach 30–50% confluence. PEDF-specific siRNA oligomers (200 pmol) and control siRNA oligomers (200 pmol) were diluted in 250 μL of OPTI-MEM I reduced-serum medium and combined with 5 μL of Lipofectamine 2000 pre-diluted in 250 μL of OPTI-MEM I. After incubation for 20 min at room temperature, the complexes were added to the cells in a final volume of 2.5 mL of medium. The human PEDF-siRNA sequences (Sigma, St Louis, MO) were as follows: 5′-CCAUCAAGCU GACUCAGGUdTdT-3′ (sense) and 5′-ACCUGAGUCAGCUUGAUGGdTdT-3′ (antisense). The negative control sequences (Sigma, St Louis, MO, USA) used were 5′-UUCUCCGAACGUGUCACGUT T-3′ (sense) and 5′-ACGUGACACGUUCGGAGAATT-3′ (antisense). After transfection for 48 h, cells were treated with 100 mg/L ox-LDL for 24 h. The silencing of PEDF was detected by western blotting and quantitative real-time PCR.

### Data analysis

Results are presented as the mean ± SD. Statistical analysis was performed using one-way analysis of variance followed by Student-Newmann-Keuls multiple comparison tests with SPSS17.0 software. For all analyses, *P*-values less than 0.05 were considered significant.

## Results

### Ox-LDL induces cytotoxicity and oxidative stress in HUVECs

**Fig. 1:**
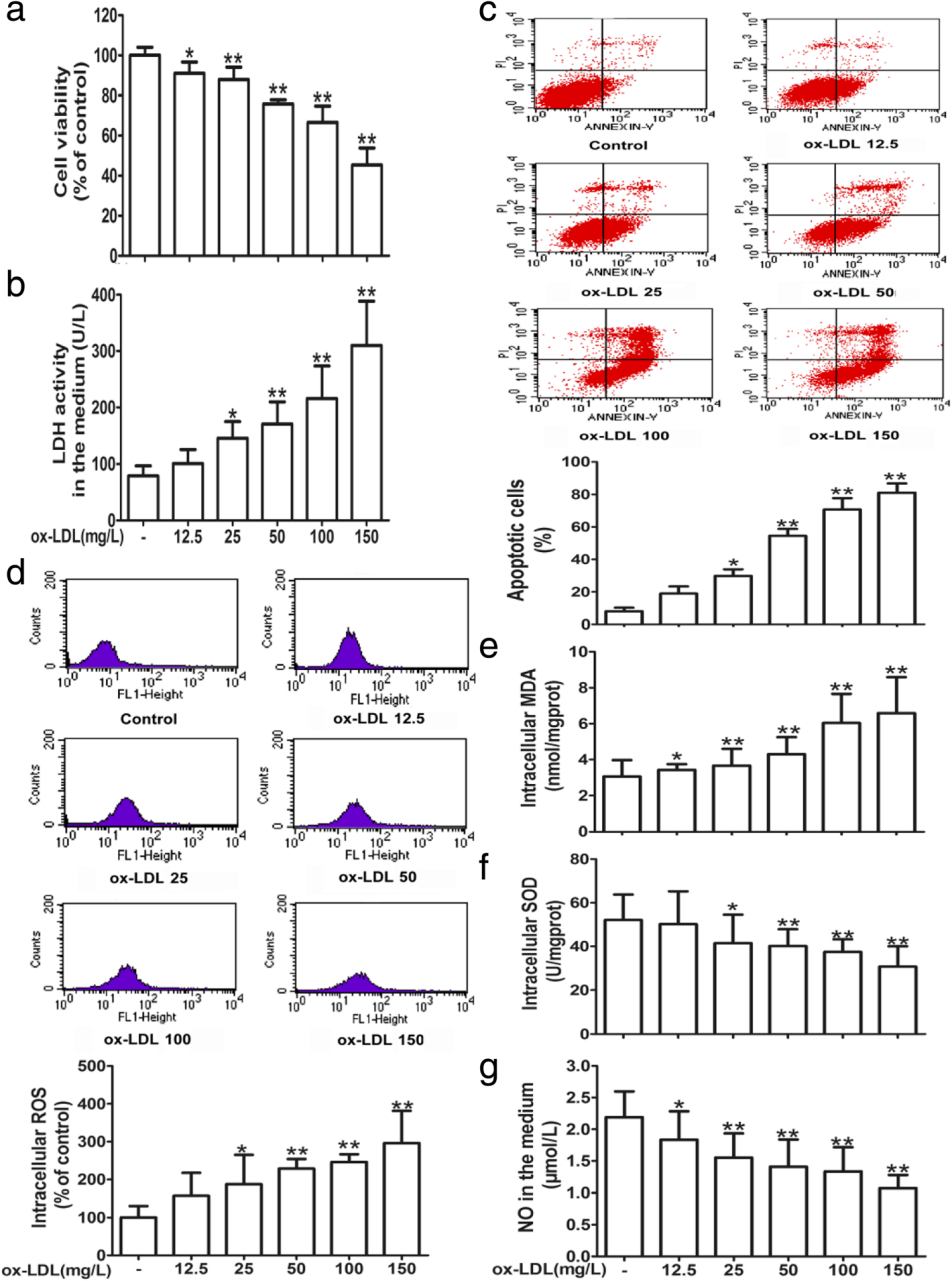
Ox-LDL induces cytotoxicity and oxidative stress in HUVECs. HUVECs were treated with ox-LDL at different concentrations (12.5, 25, 50, 100 and 150 mg/L) for 24 h. **a** Cell viability was measured by an MTT assay and results were expressed as the percentage of the control. **b** LDH activity in the medium was determined by an LDH assay kit. **c** Cell apoptosis was detected using flow cytometry, and the total apoptotic cells (early and late-stage apoptosis) are presented in the panel (Annexin V-FITC staining alone or together with PI). Intracellular ROS (**d**) generation was measured with the peroxide-sensitive fluorescent probe DCFH-DA using flow cytometry, and intracellular MDA level (**e**), SOD activity (**f**), and NO release (**g**) in the medium were detected with corresponding kits. The results are shown as the mean ± SD of at least 6 independent experiments. *P < 0.05, **P < 0.01 versus the control group

As shown in Fig. 1a and b, cell viability measured by MTT assays declined and LDH release increased in a dose-dependent manner in HUVECs treated with ox-LDL at different concentrations (12.5, 25, 50, 100 and 150 mg/L). To evaluate the inductive effect of ox-LDL on apoptosis, the proportion of apoptotic cells was quantified by Annexin V-FITC/PI double-staining (Fig. 1c) and SYTO-13/PI double-staining assays (Additional file 1: Figure S1). The results showed that treatment with ox-LDL for 24 h remarkably induced cell apoptosis in a concentration-dependent manner, compared with the control group and the native LDL (n-LDL)-treated group (Additional file 2: Figure S2).

Next, we investigated the oxidative stress generation in ox-LDL-treated cells. As illustrated in Fig. 1d, e, f and g, intracellular ROS and MDA levels were elevated in response to treatment with ox-LDL at 12.5 up to 150 mg/L for 24 h in a concentration-dependent manner. Concurrently, SOD activity was weakened and NO levels decreased in ox-LDL-treated HUVECs in a dose-dependent manner. These results indicated that ox-LDL could significantly induce HUVEC injury by triggering oxidative stress.

### Ox-LDL activates Wnt/β-catenin pathway in HUVECs

**Fig. 2:**
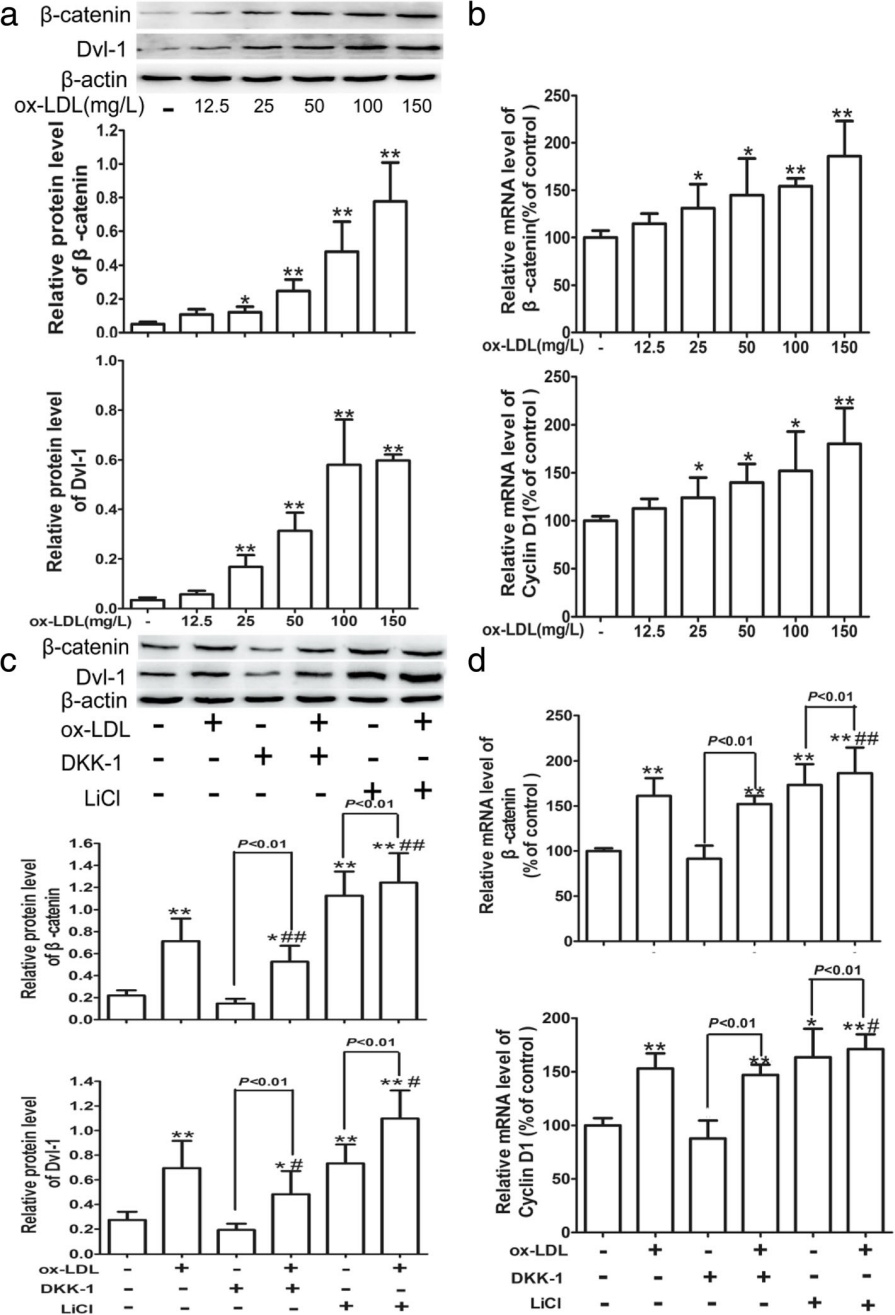
Ox-LDL activates Wnt/β-catenin signaling pathway in HUVECs. Cells were treated as described in Fig. 1, and the protein (**a**) or mRNA (**b**) levels of β-catenin, Dvl-1 and Cyclin D1 in cells were analyzed by western blotting and quantitative real-time PCR, respectively. Cells were pre-incubated with 20 nM DKK-1 or 20 mM LiCl for 24 h, followed by exposure to 100 mg/L ox-LDL for 24 h. Protein (**c**) or mRNA (**d**) levels of β-catenin, Dvl-1 and Cyclin D1 were evaluated by western blotting and quantitative real-time PCR, respectively. All data are shown as the mean ± SD of at least 3 independent experiments. *P < 0.05, **P < 0.01 versus the control group. ^#^ *P* < 0.05, ^##^ *P* < 0.01 versus the ox-LDL group. LiCl, an exogenous activator of Wnt/β-catenin pathway; DKK-1, an inhibitor of Wnt/β-catenin pathway; β-catenin, non-phosphorylated-β-catenin

The effect of ox-LDL on the expression levels of β-catenin, Dvl-1 and Cyclin D1, the hallmarks of Wnt/β-catenin pathway activation, was examined by western blotting and quantitative real-time PCR. As seen in Fig. 2a and b, ox-LDL, at concentrations of 25, 50, 100 and 150 mg/L for 24 h, increased both the protein and mRNA levels of β-catenin, the protein level of Dvl-1 and the mRNA level of Cyclin D1 in a dose-dependent manner. However, n-LDL had no significant effect on β-catenin expression (Additional file 2: Figure S2).

To further confirm the activation effect of ox-LDL on Wnt/β-catenin pathway, an exogenous Wnt/β-catenin signaling activator LiCl, and DKK-1, an inhibitor of the pathway [34], were utilized in the study. Similar to LiCl, ox-LDL resulted in a dramatic increase in the expression of β-catenin both at the protein and mRNA levels and a remarkable increase in the protein levels of Dvl-1 and the mRNA levels of Cyclin D1, and pre-incubation with LiCl synergistically promoted ox-LDL-induced up-regulation of β-catenin, Dvl-1 and Cyclin D1. However, DKK-1 pretreatment antagonized the ox-LDL-elicited up-regulation of β-catenin, Dvl-1 and Cyclin D1 (Fig. 2c and d). These findings suggested that ox-LDL may activate Wnt/β-catenin pathway in HUVECs.

### Wnt/β-catenin pathway mediates ox-LDL-induced cytotoxicity in HUVECs

**Fig. 3:**
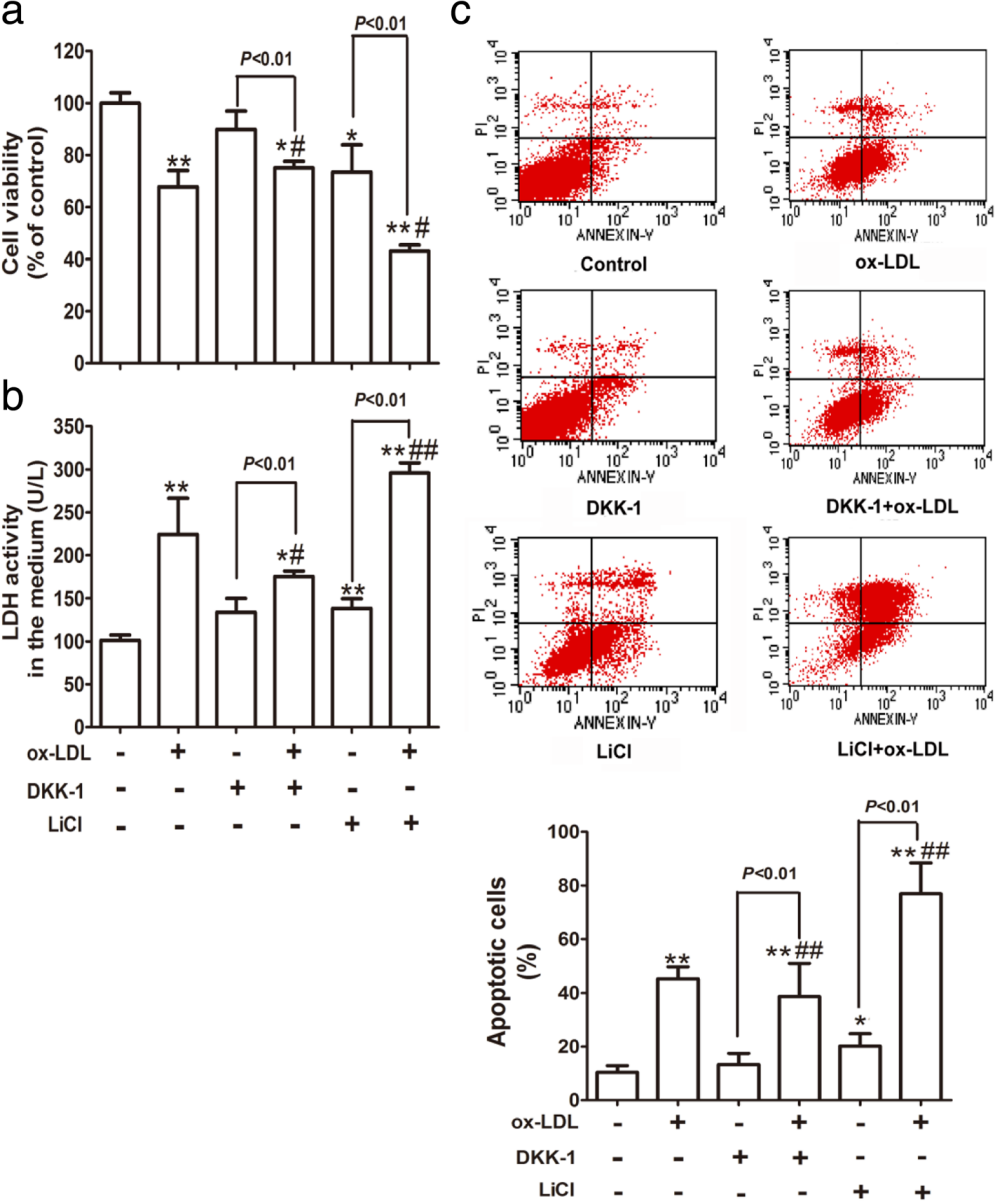
Wnt/β-catenin pathway mediates ox-LDL-induced cytotoxicity in HUVECs. HUVECs were pretreated with 20 nM DKK-1 or 20 mM LiCl for 24 h in the presence or absence of incubation with 100 mg/L ox-LDL for 24 h. Cell viability (**a**) and LDH activity in the medium (**b**) were assessed by an MTT assay and an assay kit, respectively. **c** The apoptotic cells stained with Annexin V-FITC and PI were detected by flow cytometry. All data are expressed as the mean ± SD of at least 6 independent experiments. *P < 0.05, **P < 0.01 versus the control group; ^#^ *P* < 0.05, ^##^ *P* < 0.01 versus the ox-LDL group. LiCl, an exogenous activator of Wnt/β-catenin pathway; DKK-1, an inhibitor of Wnt/β-catenin pathway

Given that ox-LDL up-regulated the expression of β-catenin and Dvl-1 in injured HUVECs, we hypothesized that Wnt/β-catenin pathway activation may be one of the underlying mechanisms of ox-LDL-induced endothelial injury. To verify this hypothesis, we measured the changes in cell viability and apoptosis in ox-LDL-incubated cells after activating or inhibiting the pathway. As seen in Fig. 3a and b, similar to LiCl, ox-LDL significantly decreased cell viability and increased the levels of LDH in the medium, effects which were jointly strengthened by LiCl pretreatment. However, pre-incubation with DKK-1 notably weakened the deleterious effect of ox-LDL on HUVECs. Similarly, the inductive effect of ox-LDL on apoptosis in HUVECs was markedly enhanced by LiCl pretreatment while pre-incubation with DKK-1 inhibited ox-LDL-induced cell apoptosis (Fig. 3c and Additional file 3: Figure S3). Cumulatively, these data revealed that Wnt/β-catenin pathway may mediate ox-LDL-induced HUVEC injury.

### Wnt/β-catenin pathway mediates ox-LDL-induced oxidative stress in HUVECs

**Fig. 4:**
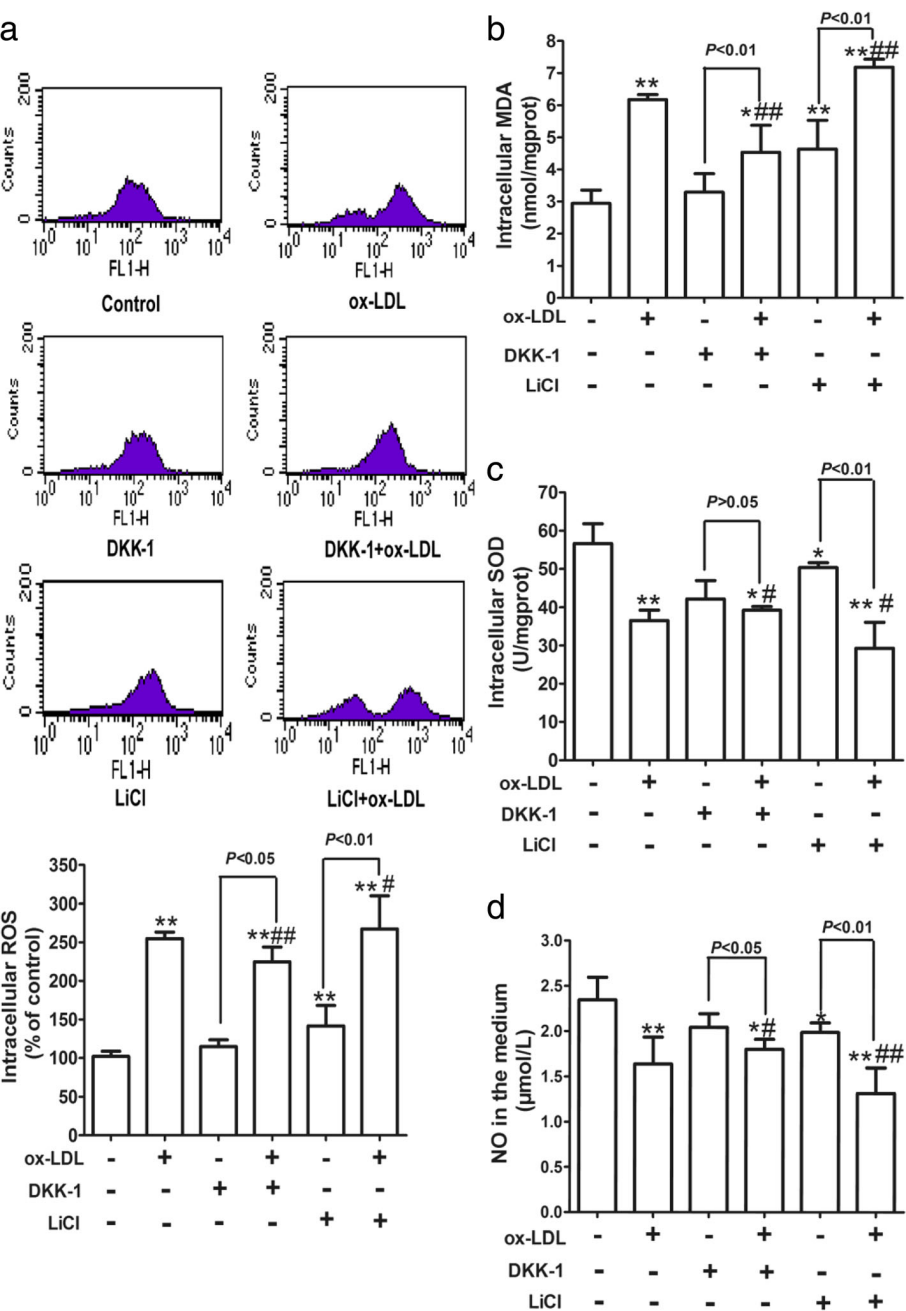
Wnt/β-catenin pathway mediates ox-LDL-induced oxidative stress in HUVECs. Cells were treated as described in Fig. 3 **a** The oxidation-sensitive fluorescent probe DCFH-DA was applied to assess intracellular ROS production by flow cytometry. MDA (**b**), SOD (**c**) and NO (**d**) levels were determined by commercial kits. The results are presented as the mean ± SD of at least 6 independent experiments. *P < 0.05, **P < 0.01 versus the control group; ^#^ *P* < 0.05, ^##^ *P* < 0.01 versus the ox-LDL group

There is evidence suggesting that activation of Wnt/β-catenin pathway is sufficient to induce retinal oxidative stress in age-related macular degeneration [37]. Therefore, whether Wnt/β-catenin pathway could be involved in ox-LDL-induced HUVEC injury by regulating oxidative stress was further explored. Figure 4 shows that, in agreement with the effect of LiCl, ox-LDL increased the levels of ROS and MDA, weakened SOD activity and reduced the NO levels. Furthermore, these effects were synergistically promoted by pretreatment with LiCl and dramatically mitigated by DKK-1 pre-incubation. These findings indicated that the Wnt/β-catenin pathway may mediate ox-LDL-induced intracellular oxidative stress in HUVECs.

### PEDF ameliorates ox-LDL-induced cytotoxicity of HUVECs

**Fig. 5:**
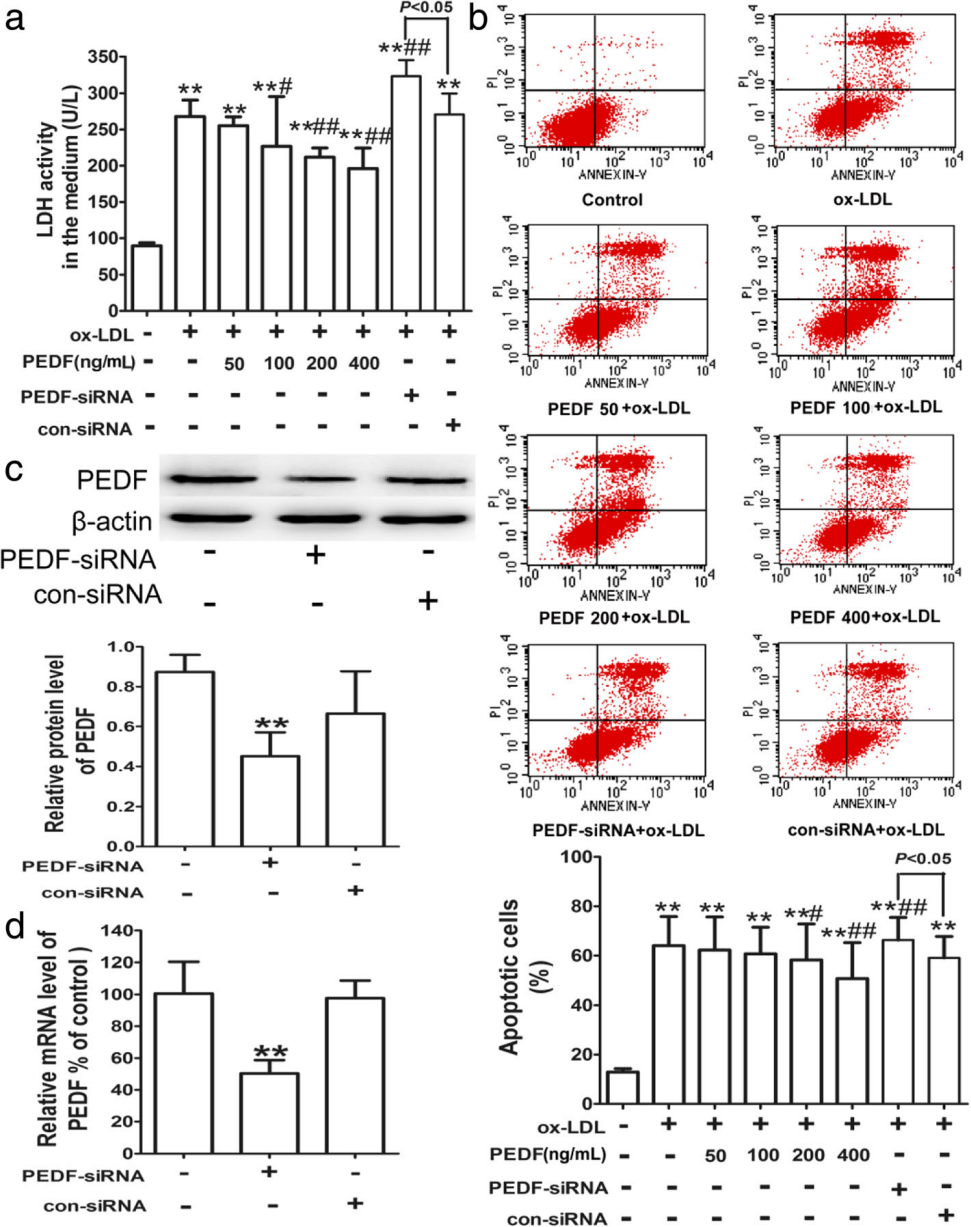
PEDF inhibits ox-LDL-induced cytotoxicity of HUVECs. HUVECs were pretreated with exogenous PEDF (50, 100, 200 and 400 ng/mL) for 24 h, or transfected with siRNA against PEDF and a negative control siRNA, followed by treatment with 100 mg/L ox-LDL for 24 h. A kit assay was performed to evaluate LDH activity (**a**) in the medium. Flow cytometry was used to analyze the ratio of apoptotic cells stained with Annexin V-FITC and PI (**b**). The silencing of PEDF was validated by western blotting (**c**) and quantitative real-time PCR (**d**). All results are presented as the mean ± SD of at least 6 independent experiments. *P < 0.05, **P < 0.01 versus the control group; ^#^ *P* < 0.05, ^##^ *P* < 0.01 versus the ox-LDL group

PEDF, a multifunctional glycoprotein, has been suggested to have anti-inflammatory, anti-oxidative, anti-thrombogenic, anti-angiogenic and anti-tumor properties. To further confirm the protective effect of PEDF on endothelial injury, HUVECs were incubated with exogenous PEDF for 24 h. As shown in Fig. 5a, b and Additional file 4: Figure S4, pretreatment with PEDF (50, 100, 200 and 400 ng/mL) led to an observable decline in LDH levels and cell apoptosis in a dose-dependent manner compared with HUVECs treated with ox-LDL alone.

Because PEDF can be secreted by many types of cells, including RPE cells, VSMCs, macrophages, ECs and cardiomyocytes, we investigated the effect of gene silencing of PEDF on ox-LDL-induced injury. PEDF-siRNA was transfected into HUVECs before incubating the cells with ox-LDL. Both protein and mRNA levels of PEDF were down-regulated by PEDF-siRNA (Fig. 5c and d). Significantly, ox-LDL-induced higher LDH levels and cell apoptosis were clearly increased in response to pre-incubation with PEDF-siRNA (Fig. 5a, b and Additional file 4: Figure S4). These data revealed that PEDF could protect HUVECs against ox-LDL-induced cytotoxicity.

### PEDF inhibits ox-LDL-induced Wnt/β-catenin pathway activation and oxidative stress in HUVECs

**Fig. 6:**
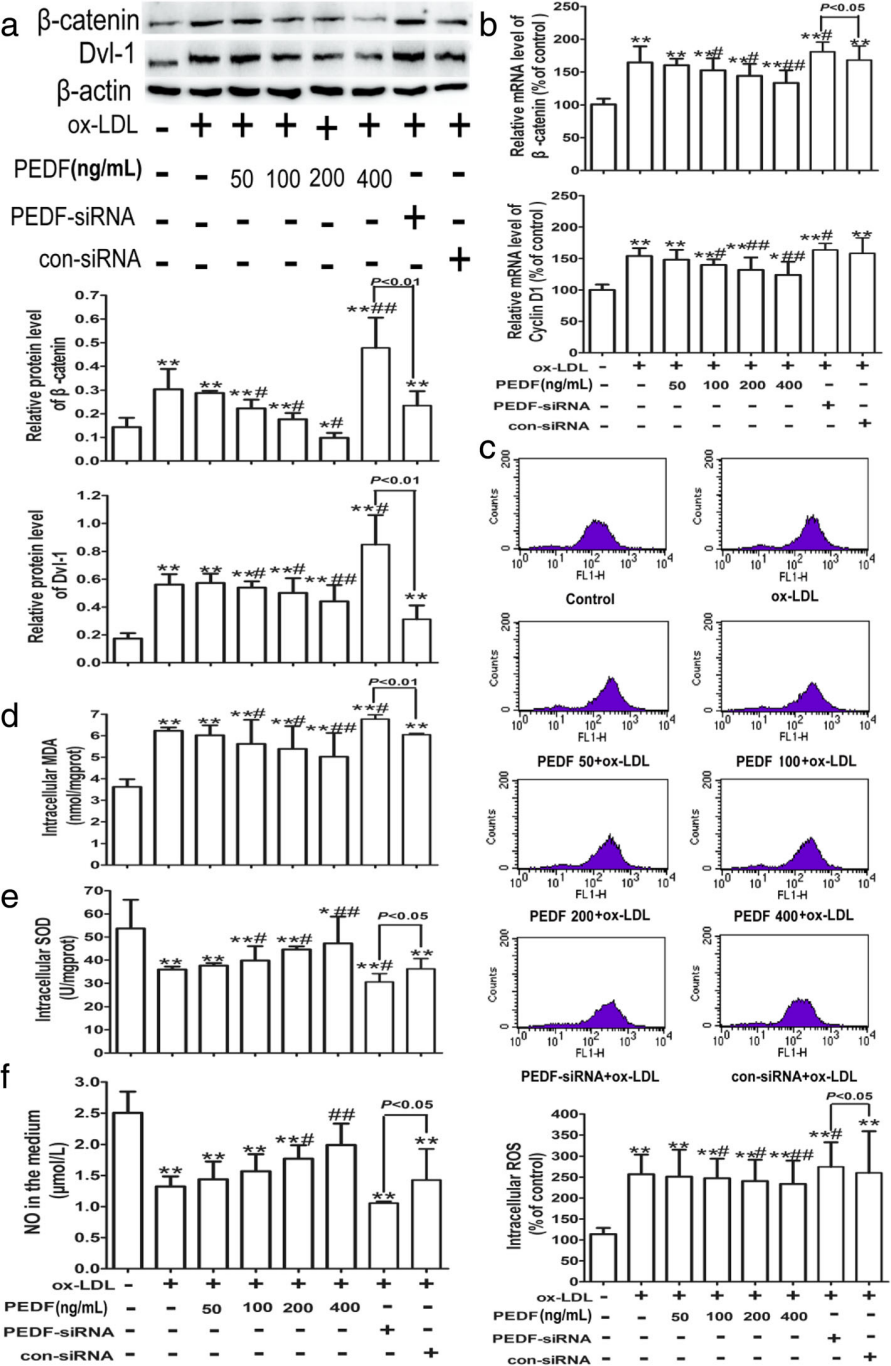
PEDF inhibits ox-LDL-induced Wnt/β-catenin pathway activation and oxidative stress in HUVECs. Cells were treated as described in Fig. 5. The protein (**a**) or mRNA (**b**) levels of β-catenin, Dvl-1 and Cyclin D1 in HUVECs were analyzed by western blotting and quantitative real-time PCR, respectively. Generation of intracellular ROS (**c**) was detected by flow cytometry using DCFH-DA as the substrate. Intracellular MDA content (**d**), SOD activity (**e**) and NO level (**f**) in the medium were tested with commercially available kits with a microplate reader according to the manufacturer’s instructions. All data are expressed as the mean ± SD of at least 3 independent experiments. *P < 0.05, **P <0.01 versus the control group; ^#^ *P* < 0.05, ^##^ *P* < 0.01 versus the 100 mg/L ox-LDL group. β-catenin, non-phosphorylated-β-catenin

Considering that PEDF has been shown to inhibit the activation of the Wnt ligand-induced Wnt pathway in retinal cells [34], we further unraveled whether PEDF could suppress Wnt/β-catenin pathway in ox-LDL-treated HUVECs. As illustrated in Fig. 6a and b, compared with the ox-LDL-treated cells, expression levels of β-catenin, Dvl-1 and Cyclin D1 dramatically declined in HUVECs pre-incubated with PEDF at concentrations of 50, 100, 200 and 400 ng/mL, while PEDF-siRNA pretreatment further increased ox-LDL-induced up-regulation of β-catenin, Dvl-1 and Cyclin D1.

Because PEDF can down-regulate intracellular production of ROS and inhibit oxidative stress, we investigated the levels of oxidative stress to determine the downstream mechanism of PEDF alleviating ox-LDL-elicited HUVEC injury by inhibiting Wnt/β-catenin pathway. Pretreatment with PEDF for 24 h observably depressed ox-LDL-induced increase in the levels of ROS and MDA and the decrease in SOD activity and NO levels, while the ox-LDL-induced oxidative stress was significantly increased by PEDF-siRNA (Fig. 6c, d, e and f). Altogether, these findings supported the idea that PEDF may block ox-LDL-induced Wnt/β-catenin pathway activation and subsequently mitigate intracellular oxidative stress.

## Discussion

In the present study, our results indicated that Wnt/β-catenin signaling pathway mediated ox-LDL-induced HUVEC injury via oxidative stress, and PEDF alleviated ox-LDL-elicited HUVEC injury by inhibiting Wnt/β-catenin signaling pathway and subsequently attenuating oxidative stress, which was supported by the following observations. First, ox-LDL activated Wnt/β-catenin signaling pathway in HUVECs. Second, similar to LiCl, ox-LDL resulted in cytotoxicity and oxidative stress in HUVECs, effects which were promoted synergistically by Wnt/β-catenin pathway activator LiCl, while they were mitigated by DKK-1, a Wnt/β-catenin pathway inhibitor. Third, pretreatment with PEDF ameliorated ox-LDL-induced cytotoxicity and oxidative stress in HUVECs and reduced the up-regulation of β-catenin, Dvl-1 and Cyclin D1 induced by ox-LDL, while this effect of ox-LDL on HUVECs was enhanced by PEDF-siRNA.

Atherosclerosis occurs in the arterial wall and is characterized by chronic lipid-triggered inflammation [2]. The increased permeability of dysfunctional endothelium leads to enhancement of vascular lipoprotein accumulation, monocyte infiltration and VSMC transmigration. Lipid accumulated in the extracellular matrix can be modified and subsequently promote a series of events in the inflammatory process. Monocytes transmigrating in the subendothelium engulf modified lipid and differentiate into foam cells. VSMCs migrating to intima synthesize extracellular matrix and promote fibrous cap formation. Thus, endothelial injury is the initial step in the procession of atherosclerosis, which can be driven by exposure to CVD risk factors. Ox-LDL can up-regulate the levels of ROS and pro-inflammatory cytokines, induce endothelial injury and promote the formation of foam cells, suggesting that it plays a key role in atherosclerosis. Our data suggested that ox-LDL significantly induced cell apoptosis, reduced cell viability and strengthened intracellular oxidative stress in HUVECs in a dose-dependent manner, which revealed the critical role of ox-LDL in endothelial injury, in agreement with previous studies.

Wnt/β-catenin signaling pathway is initiated once the Wnt protein simultaneously interacts with its receptor and co-receptor, frizzled (FZD) and LRP5/6. Immediately after binding, phosphorylation of Dvl inhibits the activity of a degradation complex consisting of adenomatous polyposis coli, axin, glycogen synthase kinase-3β and casein kinase1. As a result, β-catenin can avoid being phosphorylated and degraded, and accumulate in the cytoplasm. It then translocates into the nucleus where it interacts with the transcription factor T cell-specific transcription factor/lymphoid enhancer-binding factor (TCF/LEF). Consequently, activated Wnt/β-catenin pathway regulates the expression of downstream target genes including Cyclin D1 and c-myc, leading to many changes of pathophysiological reactions [38]. It has been shown that Wnt/β-catenin pathway is closely associated with atherosclerosis, endothelial injury, inflammation and oxidative stress [21, 37, 39]. Wnt/β-catenin pathway was found to be activated in aortic ECs of mice, and high levels of β-catenin were also found in atherosclerotic plaques in humans [12, 13]. Interestingly, ox-LDL promoted the expression of β-catenin in myofibroblasts [22] and initiated Wnt/β-catenin signaling in human RPE cells. In turn, Wnt/β-catenin pathway activation also enhanced the levels of ROS, TNF-α and VEGF [23]. Additionally, in the present study, we found that the levels of β-catenin, Dvl-1 and Cyclin D1 in HUVECs were notably increased by ox-LDL in a concentration-dependent manner, which could be synergistically enhanced by pretreatment with LiCl, an inducer of Wnt/β-catenin pathway, and remarkably antagonized by Wnt/β-catenin pathway inhibitor DKK-1. These findings indicated that ox-LDL could activate Wnt/β-catenin pathway in HUVECs. Moreover, pretreatment with LiCl further aggravated ox-LDL-driven cell death and apoptosis in HUVECs, which was blocked by DKK-1 pre-incubation, suggesting that Wnt/β-catenin pathway may mediate ox-LDL-induced HUVEC injury. In addition, there is substantial evidence confirming that suppression of Wnt/β-catenin signaling can inhibit production of ROS in bovine retinal capillary endothelial cells (RCECs) [21]. We found that in agreement with the effect of LiCl on HUVECs, treatment with ox-LDL elevated levels of ROS and MDA and reduced SOD activity and NO levels in HUVECs, and these effects were evidently enhanced by pretreatment with LiCl and attenuated by DKK-1. Altogether, the results presented above support the hypothesis that Wnt/β-catenin pathway may mediate ox-LDL-induced HUVEC injury by ptomoting oxidative stress. Besides the evidence that Wnt/β-catenin pathway could strengthen oxidative stress in the present study and other previous studies [21, 37, 39], oxidative stress has been found to activate Wnt/β-catenin pathway [19–21]. Thus, Wnt/β-catenin pathway and oxidative stress may interact with each other, which may together play an important role in the ox-LDL-induced endothelial injury and development of atherosclerosis.

PEDF is currently receiving more attention due to its anti-oxidation, anti-inflammation, and anti-thrombotic activity [26], especially in the field of atherosclerosis on the basis of the following evidence: (1) PEDF protects ECs against inflammatory activation and injury by blocking TNF-α-signals, leptin-signaling and oxidative stress induced by advanced glycation end products (AGEs) [31, 40–42]; (2) PEDF promotes plaque stability, likely by functioning as an anti-angiogenic factor in plaque angiogenesis and suppressing T cell growth and activation and SMC proliferation and migration [43–45]; (3) treatment with exogenous PEDF decreases the formation of arterial thrombi probably by inhibiting platelet activation and aggregation [46]. We previously presented evidence that D-4F relieved ox-LDL-induced endothelial injury by up-regulating PEDF. The results in the present study showed that pretreatment with PEDF ameliorated ox-LDL-induced apoptosis and cell death in a dose-dependent manner, while PEDF-siRNA enhanced ox-LDL-induced cytotoxicity, illustrating that PEDF could alleviate ox-LDL-induced endothelial injury.

The external administration of antioxidants has been demonstrated to exert the protective effects on cardiovascular disease. It has been reported that carotenoids by dietary supplementation play a protective role in atherosclerosis and CVD by promoting the activity of antioxidant system, scavenging ROS, inhibiting LDL oxidation, and protecting cellular membranes and lipoprotein against oxidative damage [47]. Similar to carotenoids, PEDF suppresses angiotensin II-induced EC activation by inhibiting nicotinamide adenine dinucleotide phosphate (NADPH) oxidase-mediated ROS generation; PEDF also inhibits the significant elevation of intracellular ROS induced by AGE-bovine serum albumin in HUVECs [31, 48], suggesting that PEDF can work as an antioxidant factor and inhibit oxidative stress. Interestingly, PEDF has also been shown to bind to LRP6 with a high affinity and block Wnt/β-catenin signaling activation in retinal cells and endothelial progenitor cells [33, 34]. Here, our results showed that the external administration of PEDF limited the increase in the protein or mRNA levels of β-catenin, Dvl-1 and Cyclin D1 in ox-LDL-treated HUVECs in a concentration-dependent manner. However, PEDF-siRNA further increased the levels of β-catenin, Dvl-1 and Cyclin D1 in ox-LDL-treated HUVECs. We also found that pre-incubation with PEDF decreased ox-LDL-induced generation of ROS and MDA, and increased SOD activity and NO levels, while the ox-LDL-induced oxidative stress was significantly strengthened by PEDF-siRNA. Taken together, these data supported the hypothesis that PEDF alleviates endothelial injury by inhibiting Wnt/β-catenin signaling and subsequently mitigating oxidative stress.

## Conclusions

The present study revealed that Wnt/β-catenin signaling pathway may mediate ox-LDL-induced endothelial injury via oxidative stress, and PEDF ameliorated endothelial injury by suppressing Wnt/β-catenin pathway and subsequently reducing the oxidative stress response. This study will add novel pieces of information regarding the mechanism of ox-LDL-induced endothelial dysfunction and application of PEDF to prevention and treatment in vascular complications. Nevertheless, there are some limitations in the present study. All data are from in vitro experiments using HUVECs and they are limited in the actual pathological process of atherosclerosis in vivo. Moreover, other test indicators closely associated with atherosclerosis, including inflammatory factors, angiogenesis and thrombosis, need be evaluated. Accordingly, further experiments need be performed to confirm these promising results.

## Additional files

Additional file 1: Figure S1. Ox-LDL induces apoptosis in HUVECs. HUVECs were treated with ox-LDL at different concentrations (12.5, 25, 50, 100 and 150 mg/L) for 24 h, and cell apoptosis was detected by SYTO-13/PI double-staining. Representative fluorescence images and quantitative data are shown. Green, SYTO-13-positive cells; red, apoptotic cells stained by PI. Scale bar =100 μm. The results are shown as the mean ± SD of 6 independent experiments. ^*^ *P* < 0.05, ^**^ *P* < 0.01 versus the control group. (PDF 243 kb)
Additional file 2: Figure S2. Effects of n-LDL and ox-LDL on cell viability, apoptosis and β-catenin expression in HUVECs. Cells were treated with n-LDL (100 mg/L) or ox-LDL (100 mg/L) for 24 h. a Cell viability was measured by an MTT assay and results were expressed as the percentage of the control. b Cell apoptosis was detected using flow cytometry, and the total apoptotic cells (early and late-stage apoptosis) are presented in the panel (Annexin V-FITC staining alone or together with PI). c Protein levels of β-catenin were evaluated by western blotting. All data are shown as the mean ± SD of at least 3 independent experiments. ***P* < 0.01 versus the control group. (PDF 237 kb)
Additional file 3: Figure S3. Wnt/β-catenin pathway mediates ox-LDL-induced apoptosis in HUVECs. HUVECs were pretreated with 20 nM DKK-1 or 20 mM LiCl for 24 h in the presence or absence of incubation with 100 mg/L ox-LDL for 24 h. The morphological changes in apoptotic cells were observed with a microscope by SYTO-13/PI double-staining. Green staining denotes fluorescence from whole cells and red staining denotes apoptotic cells visualized by PI. Representative images are shown. Scale bar = 100 μm. All data are shown as the mean ± SD of 6 independent experiments. ^*^ *P* < 0.05, ^**^ *P* < 0.01 versus the control group. ^#^ *P* < 0.05, ^##^ *P* < 0.01 versus the ox-LDL group. LiCl, an exogenous activator of Wnt/β-catenin pathway; DKK-1, an inhibitor of Wnt/β-catenin pathway. (PDF 254 kb)
Additional file 4: Figure S4. PEDF inhibits ox-LDL-induced apoptosis of HUVECs. HUVECs were pretreated with exogenous PEDF (50, 100, 200 and 400 ng/mL) for 24 h, or transfected with siRNA against PEDF and a negative control siRNA, followed by treatment with 100 mg/L ox-LDL for 24 h. Double-staining experiments show the whole cells visualized with a microscope through SYTO-13 labeling (green) and PI staining of apoptotic cells (red). Representative fluorescence images are shown (Scale bar = 100 μm). All data are expressed as the mean ± SD of 6 independent experiments. **P* < 0.05, ^**^ *P* < 0.01 versus the control group; ^#^ *P* < 0.05, ^##^ *P* < 0.01 versus the ox-LDL group. (PDF 280 kb)

## Abbreviation

ACS: Acute coronary syndrome
AGEs: Advanced glycation end products
CVD: Cardiovascular disease
DKK-1: Dickkopf 1
DMEM: Dulbecco’s modified eagle medium
DMSO: Dimethylsulfoxide
Dvl-1: Disheveled-1
ECL: Electrochemiluminescence
ECs: Endothelial cells
PEDF: Pigment epithelium-derived factor
FBS: Fetal bovine serum
FZD: Frizzled
HUVECs: Human umbilical vein endothelial cells
LDH: Lactate dehydrogenase
LiCl: Lithium chloride
LRP: Low-density lipoprotein-related protein
MDA: Malondialdehyde
NAPDH: Nicotinamide adenine dinucleotide phosphate
NO: Nitric oxide
Ox-LDL: Oxidized low- density lipoprotein
PBS: Phosphate-buffered saline
PI: Propidium iodide
PVDF: Polyvinylidene fluoride
RCEC: Retinal capillary endothelial cell
RIPA: Radio immunoprecipitation assay
ROS: Reactive oxygen species
RPE: Retinal pigment epithelium
SOD: Superoxide dismutase
TBS: Tris-buffered saline
TCF/LEF: T cell-specific transcription factor/lymphoid enhancer-binding factor
TNF: Tumor necrosis factor
VEGF: Vascular endothelial growth factor
VSMCs: Vascular smooth muscle cells
