# Supplementary figures and images for: Pigment epithelium-derived factor alleviates endothelial injury by inhibiting Wnt/β-catenin pathway
Source: Lipids Health Dis. 2017 Feb 7;16:31. doi: 10.1186/s12944-017-0407-8 (PMC5297210; doi:10.1186/s12944-017-0407-8)

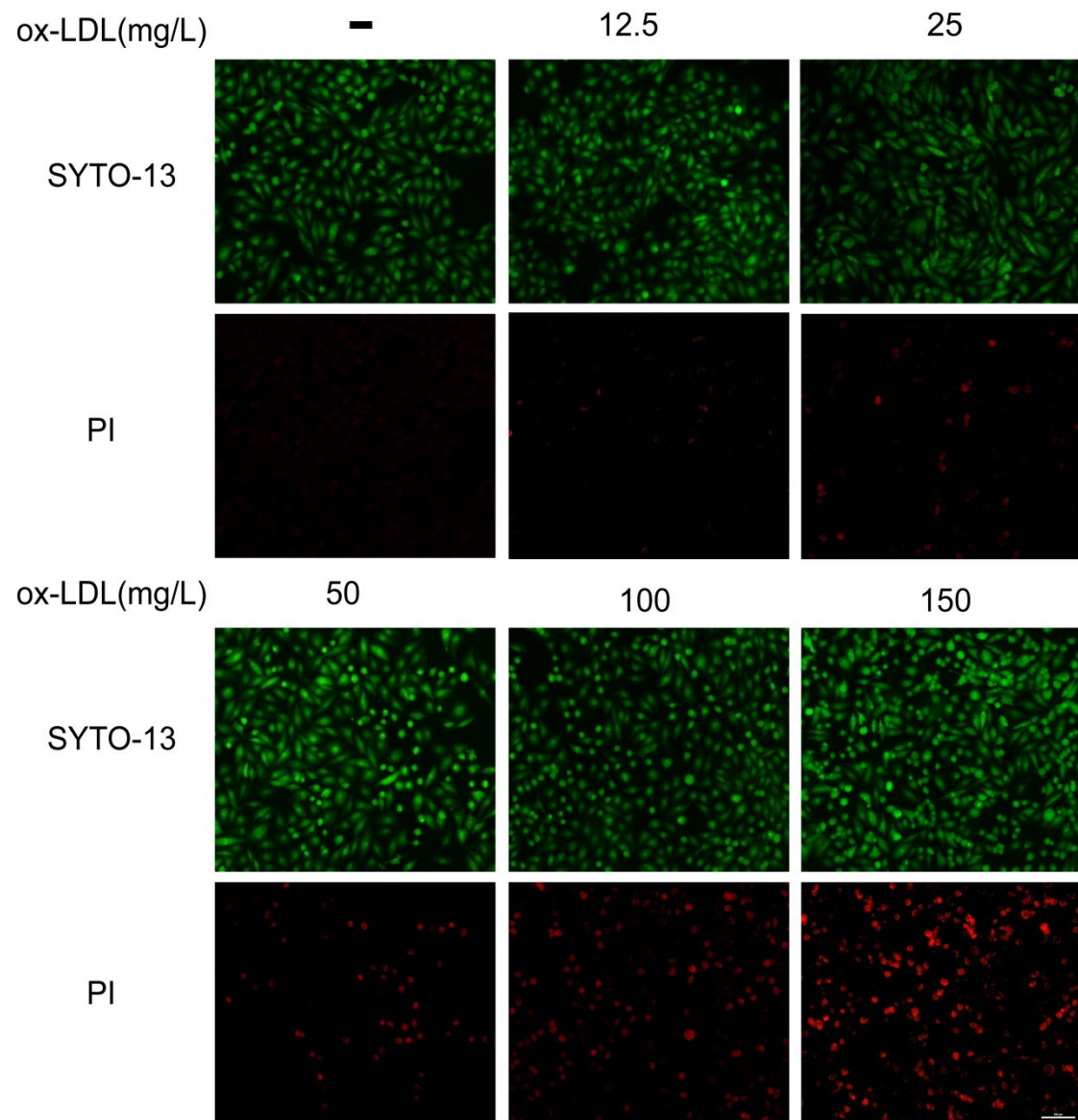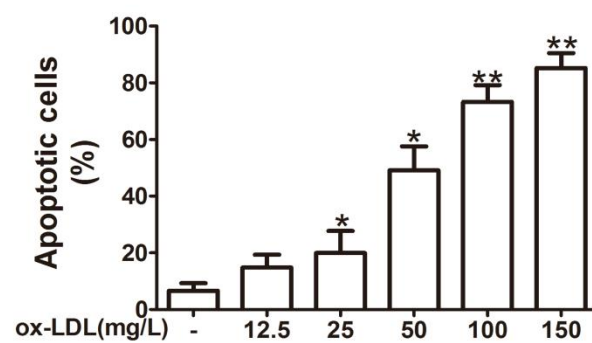

Supplement: Additional file 1: Figure S1. — Ox-LDL induces apoptosis in HUVECs. HUVECs were treated with ox-LDL at different concentrations (12.5, 25, 50, 100 and 150 mg/L) for 24 h, and cell apoptosis was detected by SYTO-13/PI double-staining. Representative fluorescence images and quantitative data are shown. Green, SYTO-13-positive cells; red, apoptotic cells stained by PI. Scale bar =100 μm. The results are shown as the mean ± SD of 6 independent experiments. * P < 0.05, ** P < 0.01 versus the control group. (PDF 243 kb) [file 12944_2017_407_MOESM1_ESM.pdf]

**a**

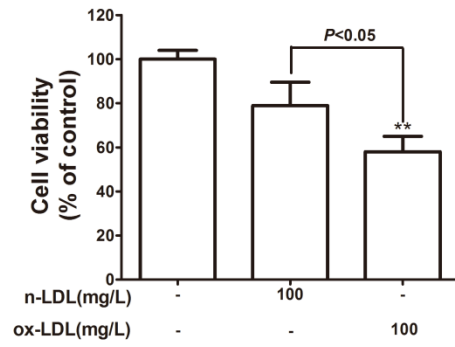

**b**

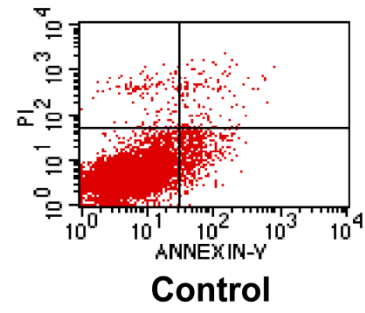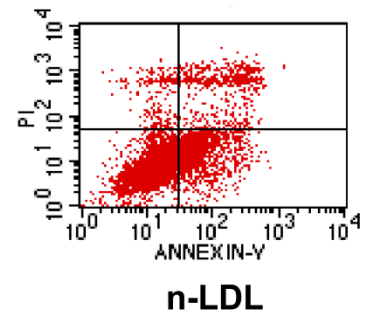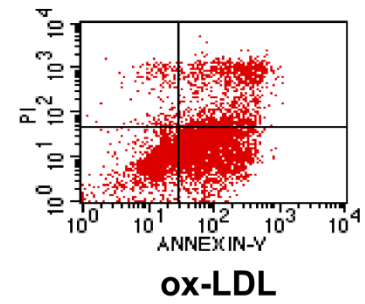

**c**

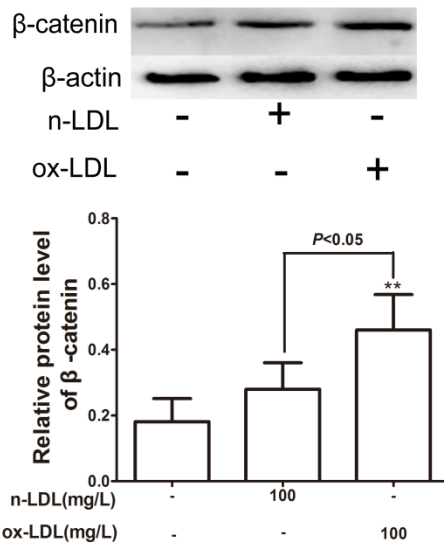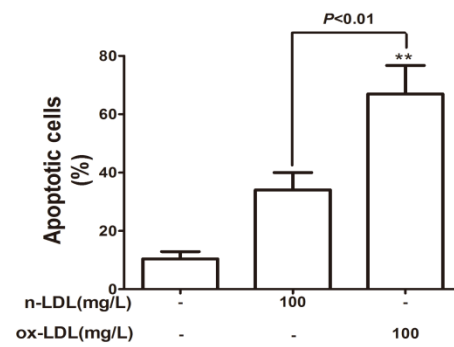

Supplement: Additional file 2: Figure S2. — Effects of n-LDL and ox-LDL on cell viability, apoptosis and β-catenin expression in HUVECs. Cells were treated with n-LDL (100 mg/L) or ox-LDL (100 mg/L) for 24 h. a Cell viability was measured by an MTT assay and results were expressed as the percentage of the control. b Cell apoptosis was detected using flow cytometry, and the total apoptotic cells (early and late-stage apoptosis) are presented in the panel (Annexin V-FITC staining alone or together with PI). c Protein levels of β-catenin were evaluated by western blotting. All data are shown as the mean ± SD of at least 3 independent experiments. **P < 0.01 versus the control group. (PDF 237 kb) [file 12944_2017_407_MOESM2_ESM.pdf]

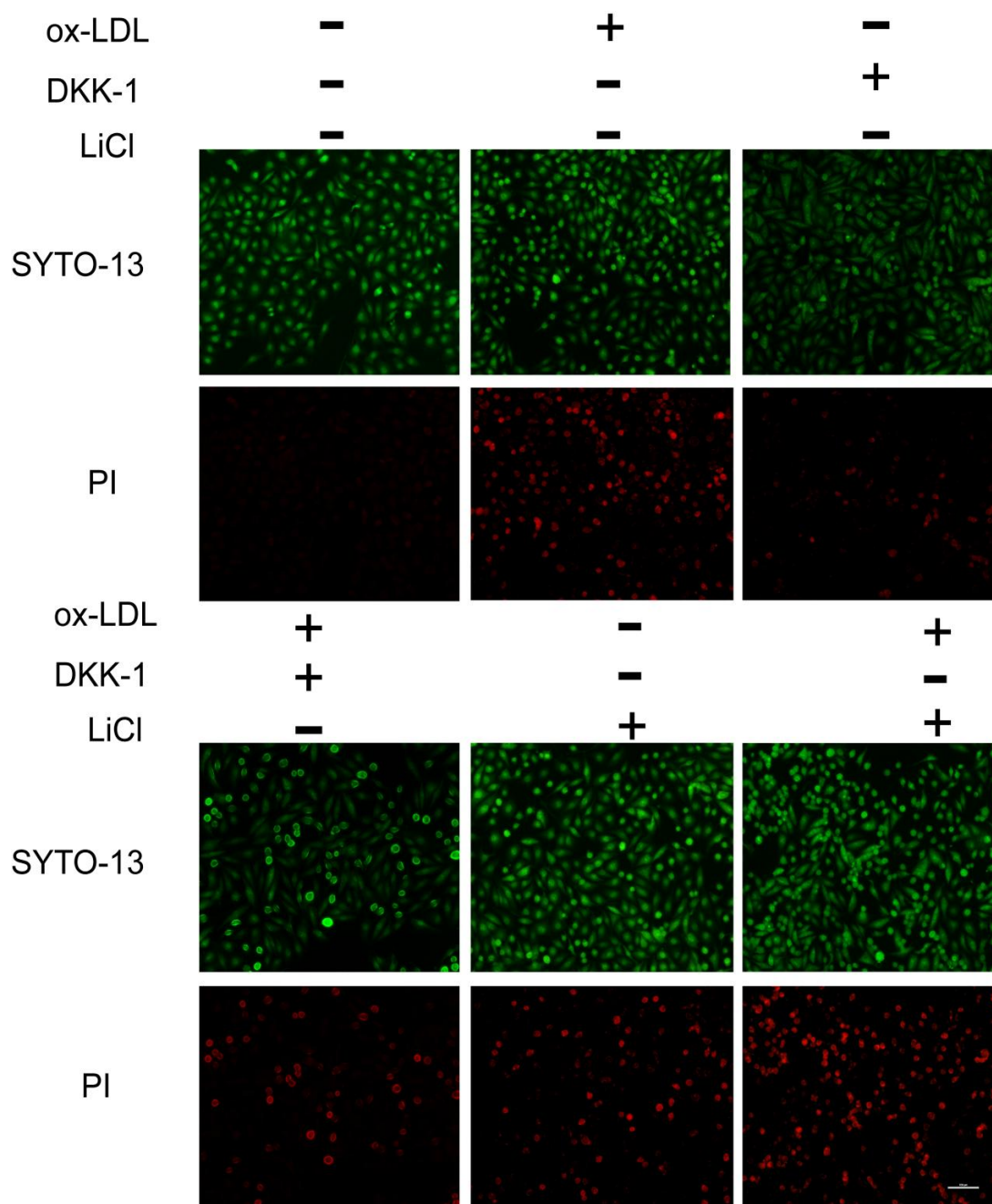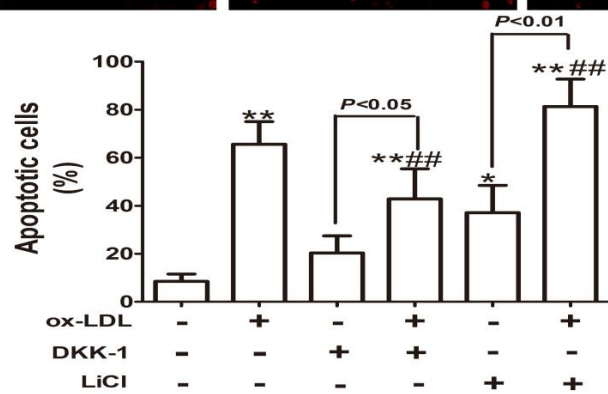

Supplement: Additional file 3: Figure S3. — Wnt/β-catenin pathway mediates ox-LDL-induced apoptosis in HUVECs. HUVECs were pretreated with 20 nM DKK-1 or 20 mM LiCl for 24 h in the presence or absence of incubation with 100 mg/L ox-LDL for 24 h. The morphological changes in apoptotic cells were observed with a microscope by SYTO-13/PI double-staining. Green staining denotes fluorescence from whole cells and red staining denotes apoptotic cells visualized by PI. Representative images are shown. Scale bar = 100 μm. All data are shown as the mean ± SD of 6 independent experiments. * P < 0.05, ** P < 0.01 versus the control group. # P < 0.05, ## P < 0.01 versus the ox-LDL group. LiCl, an exogenous activator of Wnt/β-catenin pathway; DKK-1, an inhibitor of Wnt/β-catenin pathway. (PDF 254 kb) [file 12944_2017_407_MOESM3_ESM.pdf]

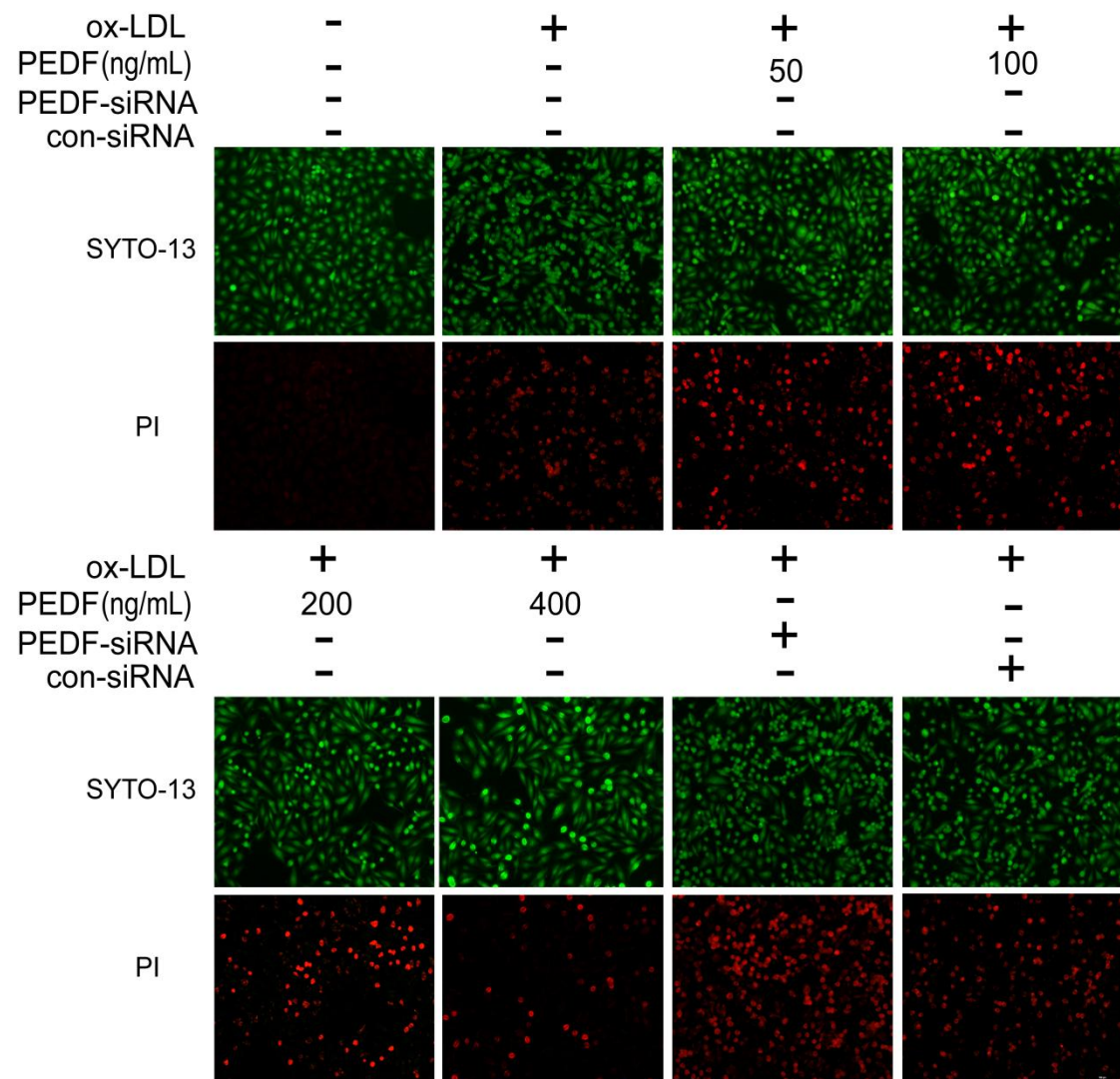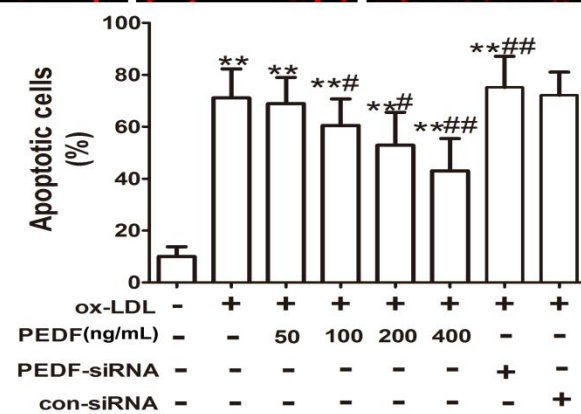

Supplement: Additional file 4: Figure S4. — PEDF inhibits ox-LDL-induced apoptosis of HUVECs. HUVECs were pretreated with exogenous PEDF (50, 100, 200 and 400 ng/mL) for 24 h, or transfected with siRNA against PEDF and a negative control siRNA, followed by treatment with 100 mg/L ox-LDL for 24 h. Double-staining experiments show the whole cells visualized with a microscope through SYTO-13 labeling (green) and PI staining of apoptotic cells (red). Representative fluorescence images are shown (Scale bar = 100 μm). All data are expressed as the mean ± SD of 6 independent experiments. *P < 0.05, ** P < 0.01 versus the control group; # P < 0.05, ## P < 0.01 versus the ox-LDL group. (PDF 280 kb) [file 12944_2017_407_MOESM4_ESM.pdf]
